# Sanitation at Sea

**Published:** 1905-05-20

**Authors:** 


### Sanitation at Sea.

There is no class of workers to which this country owes more than to the merchant seamen, and it is fitting, therefore, that their welfare should receive the first regard. Happily, public opinion is sensitive on this point, and there is every reason to hope that the disadvantages which are at the present time imposed upon the crews of many of our merchant ships will not be permitted to continue unchecked. The evils to which we refer have more than once been severely commented on in the reports of medical officers of health, but for some reason they do not appear to have attracted much general notice. We are glad, therefore, that Dr. Herbert Williams, medical officer of health for the Port of London, has again brought the matter forward, in a manner which must enforce attention, in his report for the year 1904.

Dr. Williams states that "the conditions under which seamen are compelled to exist while serving on board ship are deficient, to an extent much below that of any habitation on shore, in the requirements of air space, superficial area, ventilation and lighting." He compares the minimum amount of air space allowed for cattle in cowsheds, and for the human individual in military barracks, workrooms, lodging houses, and seamen's quarters, and shows that the cattle have the most favourable position, and the seamen occupy the worst. Details are given of four vessels constructed in recent years, and not specially selected as being among the worst examples which could be found. From these it is possible to form a definite idea of the drawbacks to which Dr. Williams refers. Thus, in one of these ships, built as recently as 1899, the forecastle, if occupied by the number of persons for which it is certified by the Board of Trade, would provide each occupant with about 85 cubic feet of air space or not much more than one-fourth of the minimum allowance in a dwelling on shore. The lighting is even less satisfactory than the air space, being only one thirty-sixth of the minimum shore allowance, so that in bright daylight, with the sun shining, it is necessary to have a lamp burning in order to read or write in the forecastle. Ventilation is nearly absent. Although the Board of Trade have laid it down that each forecastle shall be efficiently ventilated, and a cowl ventilator is placed through the deck to meet their requirements, the lower end is almost sure to be stopped up, unless, as happens more frequently, the ventilators are removed and stored in the fore peak, whilst the opening in the deck is covered securely with canvas tied on and painted.

Most people, we think, will agree with Dr. Williams that it is very wrong that men should be required to live under such conditions, and the more so in view of the fact that, as one result, pulmonary tuberculosis takes a heavy toll of the men who go down to the sea in ships.

With regard to remedies, Dr. Williams points out that the fore part of a ship is not suitable for habitation. The space is limited, and there are practical difficulties in the way of efficiently lighting and ventilating the forecastle, as the fore part of the ship is more exposed to weather. The crew therefore should be berthed aft. This arrangement of men's quarters, he states, has already been adopted by some shipowners with the result that a great improvement has been effected. He further suggests that deck houses should be provided for the crews wherever possible. And he thinks it would go some way towards remedying the present conditions if the plans of the inhabited portions of ships were always to be submitted for approval to the Port Sanitary Authority.

If we remember rightly it was urged a short while ago at a certain meeting of shipowners, that to improve the accommodation for the crews on trading ships would cost money, and would thus further handicap the British trader. It was said that the Board of Trade regulations already placed a burden on the English shipowner, for they insisted on certain requirements being observed which were expensive, and from which Scandinavian and other foreign ships were free. On these grounds the suggestion has been made, that if any further restrictions are desirable in the interests of humanity, they should be laid down by some international convention, and made to apply to all civilised countries alike, so as not to penalise unfairly those countries whose standard of hygienic morality is the highest. Whatever truth there may be in these arguments, and it must be admitted that shipowners are in the best position to judge of their value, it is to be hoped that public opinion will not be slow to realise that our sailors are not being fairly treated, and to demand that unnecessary dangers from disease shall not be added to the natural perils of the sea.

